# Risk markers of all-cause and diagnosis-specific disability pension - a prospective cohort study of individuals sickness absent due to stress-related mental disorders

**DOI:** 10.1186/1471-2458-14-805

**Published:** 2014-08-07

**Authors:** Kazi Ishtiak-Ahmed, Aleksander Perski, Ellenor Mittendorfer-Rutz

**Affiliations:** Division of Insurance Medicine, Department of Clinical Neuroscience, Karolinska Institutet, Berzelius väg 3, SE- 17177 Stockholm, Sweden; Department of Public Health Sciences, Karolinska Institutet, Stockholm, Sweden; Stockholm University, Stockholm Stress Centre, Stockholm, Sweden

**Keywords:** Stress-related mental disorders, Disability pension, Somatic disorders, Sickness absence

## Abstract

**Background:**

Stress-related mental disorders rank among the leading causes of sickness absence in several European countries. The aim of this study was to investigate predictors of all-cause and diagnosis-specific disability pension in sickness absentees with stress-related mental disorders.

**Methods:**

A cohort of 36304 non-retired individuals aged 16–64 years at 31.12.2004 with at-least one sickness absence spell due to stress-related mental disorders (SRMD) initiated in 2005 in Sweden was followed-up with regard to disability pension (2006–2010) by linkage of registers. Uni- and multivariate Hazard ratios (HR) with 95% Confidence Intervals, CI, were estimated using Cox regression for several risk markers.

**Results:**

During the follow-up period, 2735 individuals (7.5%) were granted a disability pension, predominantly due to mental diagnoses (n = 2004, 73.3%). In the multivariate analyses, female sex, age exceeding 35 years, low educational level, being born in a country outside EU25 and Northern Europe, residing outside big cities, living alone, having had a long duration of the first spell due to SRMD (>90 days); mental disorders necessitating frequent specialised health care as well as comorbid somatic disorders were found to be predictive of granting disability pension. Some different patterns emerged for risk factors related to diagnosis-specific disability pension and for younger and older individuals.

**Conclusions:**

Several predictors could be identified as risk markers for disability pension. The variation in the effect of risk markers with regard to age and diagnosis of disability pension speaks in favour of the importance of a person-centered approach in treatment and rehabilitation.

## Background

Sickness absence due to mental disorders and particularly due to stress-related mental disorders represents a major public health problem in many European countries [1]. Over the last two decades, the proportion of individuals sickness absent due to mental disorders among all sickness absentees has been increasing in most of European countries [1]. In Sweden, stress-related mental disorder (SRMDs) was one of the single most frequent sickness absence diagnoses in initiated sickness absence spells in 2012 [2]. Stress-related mental disorders correspond to the diagnostic code F43 in the International Classification of Diseases (ICD, version 10), and include reactions to severe stress and adjustment disorders [3].

Evidence suggests that the risk of granted disability pension (DP) is increased in patients sickness absent due to mental diagnoses [4–12]. Few of the existing studies, however, focused on investigating specific mental disorders [7, 13]. Disability pension as part of the social insurance system is intended to cover economical shortages due to disease and injury [14]. Still, disability pension has also been associated with adverse health behavior and social isolation [15, 16]. In order to reduce the number of individuals with mental disorders with early exit from the labour market, a recent OECD report highlights the importance of research regarding the identification of risk factors for disability pension in this group [17]. Despite the size of the public health problem of sickness absence due to stress-related mental disorders, we are not aware of any studies investigating predictors of diagnosis-specific disability pension in this group.

The study aimed to investigate the association of risk markers including socio-demographics factors, sickness absence and health care patterns to subsequent granted all-cause and diagnosis-specific DP in individuals sickness absent due to stress-related mental disorder.

## Methods

This is a prospective cohort study effected through linking of Swedish registers. The study base comprised all individuals 16 to 64 years of age at 31.12.2004 and registered in Sweden with at least one sickness absence spell due to a stress-related mental disorder (SRMD) initiated in 2005 (N = 38872). In total, 2059 and 265 individuals were excluded as they were on disability or old-age pension before baseline (2005), respectively. Two individuals were excluded as they were erroneously coded as dead before 2005. In addition, individuals with missing information on educational status (N = 41), and on the length of the first sickness absence spell due to SRMD (N = 201) were excluded. Finally, 36304 individuals (26315 women and 9,989 men) were included in the study.

Data from the following registers were used for all individuals in the study: 1) Statistics Sweden: Longitudinal integration database for health insurance and labour market studies (LISA) with socio-demographic information on age, sex, country of birth, place of residence, family situation and educational status. 2) The National Board of Health and Welfare: (i) National patient register providing information on date and diagnosis of in- and outpatient care, (ii) Cause of death register including data on date and cause of death. 3) The Social Insurance Agency (SIA): Micro-data for analyses of social insurance (MiDAS) with information on date, grade and diagnosis of sickness absence and disability pension. Linkages were based on the de-identified personal numbers of all residents in Sweden.

### Ethical considerations

This study was approved by the regional ethical review board, Karolinska Institutet, Stockholm (Dnr 2007/762-31).

### The Swedish social insurance system

In Sweden, all people above the age of 16, who live in the country and have an income from work or unemployment benefits, can receive sickness benefits if their work capacity is reduced due to disease or injury [18]. The employer pays sick pay for the first 14 days of a sick-leave spell, following a first qualifying day (without economical compensation). Thereafter, the Social Insurance Agency pays sickness benefit. Self-employed and unemployed people get all sickness benefits from the Social Insurance Agency. After seven days of self-certification, a physician certificate is required. In this study, Social Insurance Agency provided data on sick leave with benefits and data on disability pension. Individuals who have a medically certified illness or injury which contribute to a permanently reduced work capacity to at least 25% are eligible for being granted a disability pension before reaching the age for old-age retirement [19].

### Diagnostic criteria

All diagnoses related to risk factors and outcomes were based on the corresponding codes of the International Classification of Diseases (ICD) in ICD 10. Mental diagnoses were defined as F00-F99 in ICD 10. Somatic diagnoses comprised all remaining diagnoses. The definition of stress-related mental disorders was based on the corresponding ICD 10 code F43.

### Measures of risk markers

Baseline socio-demographic characteristics derived from the LISA database and measured at the end of 2004 were categorized as shown in Table 1. Both variables on sickness absence relate to spells initiated in 2005. The variable on diagnosis-specific sickness absence spells was categorized as only spells due to mental disorders; one additional spell due to a somatic disorder and multiple additional spells due to somatic disorders. Also, a variable on the numbers of sick-leave spells due to mental diagnoses (1, 2, 3+) was categorised. Duration of the first sickness absence spell due to SRMD initiated in 2005 was grouped in five categories: 1–14, 15–90, 91–180, 181–365 and more than 365 days. Categorization of health care variables was based on the diagnosis-specific median number of days and number of visits in inpatient (2000–2005) and outpatient care (2001–2005), respectively (four variables). The median length of hospital stay due to mental and somatic diagnoses was eight and four days, respectively, while the median number of visits in outpatient care due to mental and somatic diagnoses reached one and four visits, respectively.

**Table 1:** **Descriptive statistics for individuals (N = 36304) with sickness absence due to stress-related mental disorders (SRMD) initiated in 2005 and subsequent disability pension (DP, 2006–2010)**

		All	DP	DP, mental	DP, somatic
***Characteristics***	N	%	N, %	N, %	N, %
*Socio-demographic characteristics*					
Sex					
Male	9989	27.5	705 (7.1)	543 (27.1)	162 (22.2)
Female	26315	72.5	2030 (7.7)	1461 (72.9)	569 (77.8)
Age (years)					
17-25	1821	5.0	71 (3.9)	61 (3.0)	10 (1.4)
26-35	8216	22.6	313 (3.8)	261 (13.0)	52 (7.1)
36-45	11464	31.6	611 (5.3)	505 (25.2)	106 (14.5)
46-55	9138	25.2	794 (8.7)	564 (28.1)	230 (31.5)
56-65	5665	15.6	946 (16.7)	613 (30.6)	333 (45.6)
Education (years)					
0 to 9	4362	12.0	474 (10.9)	314 (15.7)	160 (21.9)
10 to 12	18066	49.8	1292 (7.2)	921 (46.0)	371 (50.8)
Above 12	13876	38.2	969 (7.0)	769 (38.4)	200 (27.4)
Country of birth*					
Sweden	31763	87.5	2264 (7.1)	1637 (81.7)	627 (85.8)
Other Nordic countries	1313	3.6	121 (9.2)	75 (3.7)	46 (6.3)
EU25 without Nordic countries	717	2.0	57 (7.9)	51 (2.5)	6 (0.8)
Rest of the world	2511	6.9	293 (11.7)	241 (12.0)	52 (7.1)
Place of residence**					
Big cities	14017	38.6	911 (6.5)	734 (36.6)	177 (24.2)
Medium sized cities	12869	35.4	1011 (7.9)	699 (34.9)	312 (42.7)
Small towns/villages	9418	25.9	813 (8.6)	571 (28.5)	242 (33.1)
Type of family^a^					
Married/cohabiting without children	4663	12.8	619 (13.3)	420 (21.0)	199 (27.2)
Married/cohabiting with children	13955	38.4	795 (5.7)	600 (29.9)	195 (26.7)
Single people without children	11490	31.6	911 (7.9)	671 (33.5)	240 (32.8)
Single people with children	6196	17.1	410 (6.6)	313 (15.6)	97 (13.3)
*Sickness absence characteristics* ^*b*^					
Number of spells					
*Only mental*	32586	89.8	2310 (7.1)	1770 (88.3)	540 (73.9)
1 spell somatic	3330	9.2	377 (11.3)	210 (10.5)	167 (22.8)
> 1 spell somatic	388	1.1	48 (12.4)	24 (1.2)	24 (3.3)
Duration (days)^c^					
1-14	10157	28.0	307 (25)	167 (8.3)	140 (19.2)
15-90	17370	47.8	575 (3.3)	338 (16.9)	237 (32.4)
91-180	3742	10.3	220 (5.9)	171 (8.5)	49 (6.7)
181-365	2469	6.8	420 (17.0)	313 (15.6)	107 (14.6)
> 365	2566	7.1	1213 (47.3)	1015 (50.6)	198 (27.1)
*Health care characteristics*					
Length of hospital stay due to mental diagnoses (days)^d^					
No hospital stay	35036	96.5	2492 (7.1)	1782 (88.9)	710 (97.1)
1 to 8	663	1.8	102 (15.4)	91 (4.5)	11 (1.5)
> 8	605	1.7	141 (23.3)	131 (6.5)	10 (1.4)
Length of hospital stay due to somatic diagnoses (days)^d^					
No hospital stay	25619	70.6	1752 (6.8)	1334 (66.6)	418 (57.2)
1 to 4	5797	16.0	528 (9.1)	374 (18.7)	154 (21.1)
> 4	4888	13.5	455 (9.3)	296 (14.8)	159 (21.8)
Outpatient care visit due to mental diagnoses^d^					
No visits	33013	90.9	2242 (6.8)	1581 (78.9)	661 (90.4)
1	1763	4.9	200 (11.3)	162 (8.1)	38 (5.2)
> 1	1528	4.2	293 (19.2)	261 (13.0)	32 (4.4)
Outpatient care visit due to somatic diagnoses^d^					
No visits	8806	24.3	462 (5.2)	356 (17.8)	106 (14.5)
1 to 4	16238	44.7	1075 (6.6)	795 (39.7)	280 (38.3)
> 4	11260	31.0	1198 (10.6)	853 (42.6)	345 (47.2)

^*^"Other Nordic countries include Denmark, Norway and Finland; EU25 comprises all countries included in the European Union in 2006 without Sweden and without "Other Nordic countries"; Rest of the world includes all remaining countries. **Place of residence: big cities: Stockholm, Gothenburg and Malmö; medium-sized cities: cities with more than 90 000 inhabitants within 30 km distance from the centre of the city ^a^Children living at home; ^b^Sickness absence due to SRMD (stress-related mental disorders);.^c^Length of the first sickness absence spell due to stress-related mental disorders; ^d^Cut-offs are based on the median for inpatient care from 2000 to 2005 and for outpatient care from 2001 to 2005.

### Outcome measure

Disability pension, was defined as granted all-cause and diagnosis-specific (mental and somatic) disability pension occurring during the follow-up period.

### Statistical analysis

Cox proportional hazard regression models were applied after assuring that the proportional hazard assumption was met. Individuals were followed from 01.01.2006 to the date of granted disability pension, death, emigration, 65 years of age or December 31^st^ 2010, whichever came first. Univariate and multivariate Hazard ratios and 95% Confidence intervals (CIs) were estimated with regard to disability pension for the various risk makers. In multivariate models all variables were mutually adjusted for each other. The partical likelihood ratio test was used to test for interaction with sex and age. Due to collinearity of the variable "number of mental sick-leave spells" with other sick-leave related covariates, a separate multivariate analysis was carried out including this variable adjusting for socio-demographics and health care determinants. All statistical analyses were conducted using SPSS software version 22.

## Results

Of the 36,304 individuals, nearly 3 times as many women (72.5%) than men (27.5%) were on sickness absence due to SRMD during 2005. The mean age of the study population was 42.9 years (standard deviation, SD: 10.7). The mean follow-up time was 4.6 years (SD:1.1). During follow-up, 317 (0.9) individuals died, while 249 (0.7) emigrated and 2380 (6.6) reached retirement age (65 years).

Descriptive statistic of individuals with a new sick-leave spell due to SRMD in 2005 and subsequent granting of disability pension is presented in Table 1. The majority of the study population had a medium level of education (50%), was born in Sweden (88%), lived in bigger cities (39%), was married/cohabiting with children living at home (38%), had only spells with mental diagnoses (90%) and a duration of the first spell of 15 to 90 days of sickness absence (48%). Most of them did not have a history of inpatient or outpatient care due to mental diagnoses (97 and 91%, respectively) or inpatient care due to somatic diagnoses (71%), but had between 1 and 4 outpatient care visits due to somatic diagnoses between 2001 and 2005 (45%).

During the five years of follow-up, 2735 individuals (7.5%) initially on sickness absence due to stress-related mental disorders were granted a disability pension (DP), the majority (n = 2004) due to mental and 731 due to somatic diagnoses. The main diagnoses for disability pension due to mental diagnoses were stress-related mental disorder (ICD 10 code, F43, N = 937, 47%) and depressive episode (ICD 10 code F32, N = 410, 20%), data not shown. The main diagnoses underlying the disability pension due to somatic diagnoses were musculoskeletal diagnoses (ICD 10 codes M00-99, N = 284, 39%), data not shown.

The proportion of individuals who were granted a disability pension during follow-up was highest among those older than 55 years (17%), with the lowest educational level (11%), those born outside of EU25 or Northern Europe (12%), living in small towns or villages (9%), those living alone (8%), and having multiple additional spells with somatic diagnoses (12%), individuals who had sick-leave spell duration exceeding one year (47%), those with more than median days/visits in in- or outpatient care due to mental (23% and 19%) and somatic diagnoses (9% and 11%), respectively.

### All-cause disability pension

Univariate and mutually adjusted HRs and 95% CIs for the various risk factors with regard to subsequent granting of disability pension are presented in Table 2. With regard to the socio-demographic characteristics, female sex, being above 35 years of age, with a low level of education (up to 9 years of schooling), born in other Nordic countries (than Sweden) or outside of Europe (EU25/Nordic countries), living outside big cities or in a family situation other than married/cohabiting with children were associated with significantly higher risks of disability pension in the univariate analyses. The risk of granted disability pension was further increased in case of one or more additional sickness absence spells due to somatic diagnoses and if the duration of the first sickness absence spell due to SRMD exceeded 3 months. The HRs and 95% CIs for one and more additional spells due to mental disorders were 1.36 (1.19-1.55) and 1.89 (1.28-2.78), respectively. The risk was also elevated with increasing number of days in in- and outpatient care due to mental or somatic diagnoses.

**Table 2:** **Unadjusted and multivariate adjusted hazard ratios (HR) with 95% confidence intervals, CI, for disability pension (DP) in individuals (N = 36 304) with sickness absence due to stress related mental diagnoses (SRMD) initiated in 2005**

***Characteristics***	Crude HR	CI 95%	Adjusted HR	CI 95%)
*Socio-demographic characteristics*				
Sex				
Female	1.09	1.00-1.89	1.15	1.05-1.25
Male	1		1	
Age (years)				
17-25	1		1	
26-35	0.98	0.75-1.26	1.09	0.84-1.42
36-45	1.38	1.08-1.76	1.64	1.27-2.11
46-55	2.29	1.79-2.91	2.81	2.19-3.60
56-65	5.28	4.15-6.72	5.30	4.15-6.83
Education (years)				
0 to 9	1.63	1.46-1.82	1.33	1.18-1.48
10 to 12	1.02	0.94-1.11	1.06	0.98-1.16
Above 12	1		1	
Country of birth*				
Sweden	1		1	
Other Nordic countries	1.33	1.11-1.60	1.16	0.96-1.40
EU25 without Nordic countries	1.14	0.88-1.48	0.87	0.66-1.13
Rest of the world	1.67	1.48-1.89	1.75	1.54-1.99
Place of residence**				
Big cities	1		1	
Medium sized cities	1.22	1.12-1.34	1.16	1.06-1.27
Small town	1.35	1.23-1.48	1.33	1.21-1.47
Type of family				
Married/cohabiting with children^a^	1		1	
Married/cohabiting without children^a^	2.60	2.34-2.89	1.21	1.07-1.36
Single without children^a^	1.45	1.32-1.60	1.19	1.08-1.32
Single with children^a^	1.17	1.04-1.32	1.02	0.90-1.15
*Sickness absence characteristics* ^*b*^				
Number of spells				
*Only mental*	1		1	
1 spell somatic	1.65	1.47-1.83	1.44	1.28-1.61
> 1 spell somatic	1.80	1.36-2.24	1.78	1.32-2.34
Duration (days)^c^				
1-14	1		1	
15-90	1.10	0.96-1.26	1.11	0.97-1.28
91-180	1.99	1.68-2.37	1.99	1.66-2.35
181-365	6.24	5.38-7.23	5.90	5.09-6.84
> 365	20.72	18.3-23.5	16.57	14.6-18.8
*Health care characteristics*				
Length of hospital stay due to mental diagnoses (days)^d^				
No hospital stay	1		1	
1 to 8	2.29	1.88-2.79	1.46	1.19-1.79
> 8	3.59	3.03-4.26	1.78	1.48-2.15
Length of hospital stay due to somatic diagnoses (days)^d^				
No hospital stay	1		1	
1 to 4	1.34	1.22-1.48	1.06	0.96-1.17
> 4	1.38	1.25-1.53	1.08	0.97-1.20
Outpatient care visit due to mental diagnoses (days)^d^				
No visits	1		1	
1	1.69	1.46-1.96	1.29	1.12-1.50
>1	3.04	2.69-3.44	1.52	1.32-1.74
Outpatient care visit due to somatic diagnoses (days)^d^				
No visits	1		1	
1-4	1.27	1.14-1.42	1.16	1.04-1.30
> 4	2.09	1.88-2.33	1.51	1.35-1.71

^*^"Other Nordic countries include Denmark, Norway and Finland; EU25 includes all countries included in the European Union in 2006 without Sweden and without "Other Nordic countries"; Rest of the world includes all remaining countries. **Place of residence: big cities: Stockholm, Gothenburg and Malmö; medium-sized cities: cities with more than 90 000 inhabitants within 30 km distance from the centre of the city; ^a^Children living at home; ^b^Sickness absence due to SRMD (stress related mental diagnoses); ^c^Length of the first sickness absence spell due to stress related mental diagnoses; ^d^Cut-offs are based on the median, inpatient care from 2000–2005; outpatient care from 2001–2005. Adjusted HRs indicate that all variables have been mutually adjusted for each other.

In the multivariate analysis, mutually adjusting for all risk markers, female sex, low educational level, country of birth outside Europe (EU25/Nordic countries), living outside big cities as well as living without children remained to be significant predictors. The risk of disability pension increased with increasing age at baseline. Individuals of more than 55 years of age had a much higher HR (5.30, 95% CI 4.15-6.83) compared to those below 25 years of age. The risk increased with rising number of spells due to somatic diagnoses and with increasing duration of the first spell in the multivariate analysis. Also the risk associated with one additional mental sick-leave spell remained higher after multivariate adjustment, HR 1.17, CI 1.02-1.33. Inpatient care due to mental diagnoses and outpatient care due to mental and somatic diagnoses were signficantly associated with an increased risk of subsequent disability pension, regardless of length of hospital stay or number of visits.

### Disability pension due to mental and somatic diagnoses

Table 3 shows multivariate adjusted Hazard ratios and 95% Confidence Intervals for the various risk markers in relation to granted DP due to mental and somatic diagnoses, separately. Female sex and low educational level turned out to be significant predictors for DP due to somatic diagnoses, while living alone was predictive for DP due to mental diagnoses. The risk of granted DP increased with increasing age with higher risk estimates related to DP due to somatic diagnoses than due to mental diagnoses. For example, the HRs related to the age group 56 to 65 years (reference group comprised those between 17 and 25 years of age) for DP due to mental diagnoses and due to somatic diagnoses were 3.76, 95% CI 2.86-4.95 and 14.59, 95% CI 7.70-27.63, respectively (Table 3). Being born outside Europe (Northern Europe/EU25) turned out to be a similarly important risk marker for DP due to mental and somatic diagnoses, while being born in other Nordic countries than Sweden was only predictive of DP due to somatic diagnoses. Residing in small towns or villages as compared to big cities was associated with higher risk estimates for DP due to somatic than mental diagnoses.

**Table 3:** **Multivariate adjusted hazard ratios (HR) with 95% confidence intervals, CI, for disability pension (DP) due to mental and somatic diagnoses in individuals (N = 36 304) with sickness absence due to stress related mental diagnoses (SRMD) initiated in 2005**

	DP mental diagnoses		DP somatic diagnoses	
***Characteristics***	Adjusted hazard ratios (HR)	Confidence interval (CI 95%)	Adjusted hazard ratios (HR)	Confidence interval (CI 95%)
*Socio-demographic characteristics*				
Sex				
Female	1.07	0.97-1.19	1.36	1.14-1.63
Male	1		1	
Age (years)				
17-25	1		1	
26-35	1.03	0.78-1.37	1.32	0.67-2.61
36-45	1.49	1.13-1.97	2.17	1.12-4.19
46-55	2.20	1.67-2.89	6.08	3.20-11.53
56-65	3.76	2.86-4.95	14.59	7.70-27.63
Education (years)				
0 to 9	1.10	0.96-1.26	2.14	1.73-2.64
10 to 12	0.94	0.86-1.04	1.53	1.29-1.82
Above 12	1		1	
Country of birth*				
Sweden	1		1	
Other Nordic countries	1.00	0.79-1.27	1.51	1.12-2.05
EU25 without Nordic countries	1.03	0.78-1.36	0.39	0.17-0.87
Rest of the world	1.76	1.53-2.03	1.60	1.19-2.14
Place of residence**				
Cities	1		1	
Medium sized cities	0.98	0.89-1.11	1.84	1.53-2.22
Small towns/villages	1.18	1.06-1.32	1.95	1.60-2.38
Type of family				
Married/cohabiting with children^a^	1		1	
Married/cohabiting without children^a^	1.22	1.06-1.39	1.14	0.91-1.42
Single people without children^a^	1.17	1.04-1.31	1.19	0.97-1.46
Single people with children^a^	1.04	0.91-1.19	0.98	0.77-1.26
*Sickness absence characteristics* ^*b*^				
Number of spells				
*Only mental*	1		1	
1 spell somatic	1.11	0.96-1.28	2.31	1.93-2.75
> 1 spell somatic	1.25	0.84-1.88	2.97	1.97-4.49
Duration (days)^c^				
1-14	1		1	
15-90	1.19	0.99-1.43	1.03	0.84-1.27
91-180	2.76	2.23-3.42	1.04	0.75-1.44
181-365	7.89	6.54-9.53	3.53	2.74-4.54
> 365	24.99	21.17-29.51	6.50	5.22-8.11
*Health care characteristics*				
Length of hospital stay due to mental diagnoses (days)^d^				
No hospital stay	1		1	
1 to 8	1.68	1.35-2.10	0.66	0.36-1.20
> 8	2.03	1.67-2.47	0.71	0.37-1.36
Length of hospital stay due to somatic diagnoses (days)^d^				
No hospital stay	1		1	
1 to 4	0.99	0.87-1.12	1.31	1.08-1.59
> 4	0.92	0.80-1.05	1.58	1.30-1.93
Outpatient care visits due to mental diagnoses (days)^d^				
No visits	1		1	
1	1.41	1.19-1.66	1.05	0.75-1.46
>1	1.68	1.44-1.95	0.96	0.65-1.39
Outpatient care visits due to somatic diagnoses (days)^d^				
No visits	1		1	
1-4	1.11	0.98-1.26	1.33	1.06-1.67
> 4	1.38	1.21-1.58	1.98	1.56-2.51

^*^"Other Nordic countries include Denmark, Norway and Finland; EU25 includes all countries included in the European Union in 2006 without Sweden and without "Other Nordic countries"; Rest of the world includes all remaining countries. **Place of residence: big cities: Stockholm, Gothenburg and Malmö; medium-sized cities: cities with more than 90 000 inhabitants within 30 km distance from the centre of the city; ^a^Children living at home; ^b^Sickness absence due to SRMD (stress related mental diagnoses); ^c^Length of the first sickness absence spell due to stress related mental diagnoses; ^d^Cut-offs are based on the median, inpatient care from 2000–2005; outpatient care from 2001–2005. Adjusted HRs indicate that all variables have been mutually adjusted for each other.

With regard to the sickness absence characteristics, increasing duration of the first sickness absence spell due to SRMD was both strongly related to the risk of DP due to mental and somatic diagnoses, considerably stronger to DP due to mental diagnoses, though. Having had more than half a year of sickness absence was associated with a HR 7.89, 95% CI 6.54-9.53 and HR 3.53, 95% CI 2.74-4.54 for DP due to mental and somatic diagnoses compared to having absences up to 2 weeks, respectively. Also, having at least one spell due to somatic diagnoses in addition to the spell due to SRMD, was only predictive of DP due to somatic diagnoses, not of DP due to mental diagnoses. These differences were also noted for in- and specialised outpatient health care due to mental and somatic diagnoses. In- and specialised outpatient care due to mental diagnoses was only associated with a significantly higher risk of granted DP due to mental diagnoses, but not due to somatic diagnoses. The opposite was true for in- and outpatient care due to somatic diagnoses, which was only predictive of DP due to somatic diagnoses, with one exception. The HR for having had more than the median outpatient care visits due to somatic diagnoses (2001–05) was 1.38, 95% CI 1.21-1.58 for DP due to mental diagnoses compared to having had no such outpatient care.

The partial likehood ratio test revealed significant interactions between the covariates and age. Analyses have therefore been stratified for age. Multivariate HRs and 95% CIs related to the various covariates for individuals below and above 45 years of age are shown in Table 4. Female sex, low education, residing in small towns or villages, sick-leave duration exceeding one year and inpatient health care due to mental disorders seemed to be more strongly associated with subsequent granting of disability pension in individuals younger than 45 years than for their older counterparts. On the other hand, living alone or living with a partner but without children and having somatic sick-leave spells were associated with higher HRs for the older cohort.

**Table 4:** **Multivariate adjusted hazard ratios (HR) with 95% confidence intervals, CI, for disability pension (DP) in individuals (N = 36 304) with sickness absence due to stress-related mental diagnoses (SRMD) initiated in 2005, stratified for age (<= and > 45 years)**

	<= 45 years		> 45 years	
***Characteristics***	Adjusted hazard ratios (HR)	Confidence interval (CI 95%)	Adjusted hazard ratios (HR)	Confidence interval (CI 95%)
*Socio-demographic characteristics*				
Sex				
Female	1.26	1.07-1.47	1.03	0.92-1.15
Male	1		1	
Education (years)				
0 to 9	1.47	1.21-1.78	1.22	1.06-1.40
10 to 12	1.06	0.92-1.23	0.99	0.90-1.11
Above 12	1		1	
Country of birth*				
Sweden	1		1	
Other Nordic countries	1.36	0.95-1.56	1.11	0.89-1.37
EU25 without Nordic countries	1.04	0.63-1.71	0.83	0.61-1.14
Rest of the world	1.59	1.34-1.89	1.78	1.48-2.14
Place of residence**				
Cities	1		1	
Medium sized cities	1.18	1.01-1.37	1.14	1.01-1.27
Small towns/villages	1.56	1.33-1.84	1.19	1.05-1.35
Type of family				
Married/cohabiting with children^a^	1		1	
Married/cohabiting without children^a^	0.82	0.57-1.18	1.73	1.52-1.97
Single people without children^a^	1.06	0.91-1.23	1.49	1.31-1.71
Single people with children^a^	1.02	0.86-1.20	1.01	0.84-1.19
*Sickness absence characteristics* ^*b*^				
Number of spells				
*Only mental*	1		1	
1 spell somatic	1.29	1.07-1.56	1.57	1.37-1.81
> 1 spell somatic	1.09	0.63-1.90	2.33	1.66-3.26
Duration (days)^c^				
1-14	1		1	
15-90	1.24	0.97-1.59	1.08	0.91-1.28
91-180	1.81	1.32-2.48	2.12	1.73-2.61
181-365	5.33	4.06-7.01	6.44	5.40-7.67
> 365	23.04	18.35-28.91	15.04	12.89-17.56
*Health care characteristics*				
Length of hospital stay due to mental diagnoses (days)^d^				
No hospital stay	1		1	
1 to 8	1.83	1.41-2.39	1.01	0.72-1.43
> 8	2.10	1.70-2.78	1.43	1.08-1.90
Length of hospital stay due to somatic diagnoses (days)^d^				
No hospital stay	1		1	
1 to 4	1.09	0.92-1.28	1.02	0.89-1.16
> 4	1.08	0.91-1.28	1.03	0.89-1.19
Outpatient care visits due to mental diagnoses (days)^d^				
No visits	1		1	
1	1.17	0.94-1.47	1.33	1.09-1.62
>1	1.51	1.25-1.83	1.30	1.05-1.60
Outpatient care visits due to somatic diagnoses (days)^d^				
No visits	1		1	
1-4	1.07	0.88-1.30	1.23	1.08-1.41
> 4	1.52	1.24-1.86	1.53	1.32-1.77

^*^"Other Nordic countries include Denmark, Norway and Finland; EU25 includes all countries included in the European Union in 2006 without Sweden and without "Other Nordic countries"; Rest of the world includes all remaining countries. **Place of residence: big cities: Stockholm, Gothenburg and Malmö; medium-sized cities: cities with more than 90 000 inhabitants within 30 km distance from the centre of the city; ^a^Children living at home; ^b^Sickness absence due to SRMD (stress-related mental diagnoses); ^c^Length of the first sickness absence spell due to stress-related mental diagnoses; ^d^Cut-offs are based on the median, inpatient care from 2000–2005; outpatient care from 2001–2005. Adjusted HRs indicate that all variables have been mutually adjusted for each other.

## Discussion

### Main findings

In this population based cohort study of individuals with a new sickness absence spell due to stress-related mental disorders in 2005, being female, over 35 years of age, with low educational level, having been born outside of Europe (EU25/Nordic countries), living outside big cities or alone were found to be predictors of granted all-cause disability pension during the five subsequent years of follow-up. In addition, long duration of sick-leave spells due to SRMD, additional spells and outpatient care due to somatic diagnoses as well as in- and specialised outpatient care due to mental diagnoses predicted all-cause DP. Female sex, low educational level, living outside big cities, sickness absence spells and inpatient care due to somatic diagnoses turned out to be only predictive of DP due to somatic diagnoses. On the other hand, living alone as well as in- and outpatient care due to mental diagnoses were associated with higher risks for DP due to mental diagnoses.

### Methodology

Among the strengths of this study the large study population consisting of 36 546 individuals with a new sickness absence due to SRMD, practically no drop out with a long follow-up of five years and the use of data of good quality should be mentioned [20–22]. The study had further the opportunity to examine a wide variety of socio-demographic factors as well as factors related to sickness absence and health care patterns.

The validity of sickness absence and disability pension diagnoses is often discussed. A previous Swedish study, however, assessed the validity of sickness absence diagnoses and considered it to be good [21]. Moreover, there is an on-going debate regarding the differentiation between stress-related mental disorders and depression [23]. Still, diagnostic guidelines for stress-related mental disorders published by the National Board of Health and Welfare in Sweden determine that in the presence of a depression, depression should be used as the main diagnosis [23]. Moreover, previous research suggests that individuals sickness absent due to stress-related mental disorders differ considerably from the individuals absent due to depression with regard to their risk of disability pension and the degree of previous health-care consumption [24].

We may have missed individuals with a stress-related mental disorder as a secondary diagnosis, as in the Social Insurance Agency’s register only the main diagnosis was recorded for any sickness absence spell. Also any comorbid disorders not covered by the information in the in- and specialised outpatient care register may have been missed. We therefore anticipate that only mental and somatic disorders of greater medical severity were covered. This study investigated a wide range of different risk factors for disability pension in individuals sickness absent due to stress-related mental disorders. Still, additional risk factors, like e.g. overweight, might have an association with subsequent labor market exit and may have an effect on the estimates of the studied covariates [25]. However, such information was not available in the database used.

We found that female sex, increasing age, low education and living in small towns/villages were associated with an increased risk of disability pension. These findings are consistent with the findings of previous studies of individuals on long term sickness absence due to mental disorders and individuals with outpatient care due to depression [6, 26]. Analyses of diagnosis-specific DP revealed that low education was not associated with an increased risk for DP due to mental diagnoses. These findings are comparable to results from a previous study [26]. On the other hand, low education was clearly predictive of DP due to somatic disorders. Further studies are warranted to scrutinise these relations with regard to additional determinants of socio-economic status and specific somatic diagnoses of DP.

Being born in a country outside Northern Europe and outside EU25 was predictive for DP, both due to mental and somatic diagnoses. The finding that first generation immigrants have a higher risk for disability pension was earlier reported in studies based on the general population [27] and individuals on long term sick-leave [12], but to our best knowledge not on individuals sickness absent due to SRMD. Previously, differences in health care access and utilisation as well as working conditions and income in first generation immigrants compared to the native population have been reported [28]. In our analyses, we controlled for differences in specialised health care, socio-economic status and a range of additional socio-demographic factors. Future studies are warranted in order to elucidate mechanisms behind the higher risk of disability pension among immigrants with sickness absence due to SRMD, particularly those with a birth country outside Europe (EU25/Nordic countries).

The risk of being granted disability pension was found to increase with increasing duration of sick leave and number of mental sick-leave spells, which is consistent with previous reports based on the general population and occupational cohorts [10, 11, 29–31]. Duration of sickness absence and several spells may not solely be a measure of the chronicity and severity of the underlying disorder [32], but might also be a marker of inadequate treatment and rehabilitation efforts [32]. Moreover, increasing duration of sickness absence might be associated with social isolation and increased alcohol consumption [33], which in turn may increase the risk of disability pension. In contrast to the association of sickness absences exceeding three months with disability pension, sickness absence shorter than three months was not associated with an increased risk of DP. Future studies are needed to investigate the optimal, individually varying duration of sickness absence due to stress-related mental disorders in order to prevent early exit from the labour market.

Frequent and long duration of specialised health care due to mental diagnoses predicted all-cause DP and DP due to mental disorders, but not DP due to somatic disorders. These findings are consistent with previous studies, indicating that frequent health care consumption was related with an increased risk of DP [34]. Comorbid somatic disorders, measured as additional sickness absence spells and health care due to somatic diagnoses, were risk factors for all-cause DP, particularly for DP due to somatic disorders. Further research is required to evaluate possible implication of these findings for treatment and rehabilitation after sick-leave due to stress-related mental disorders.

We found that the effect of risk markers varied with age. To the best of our knowledge, this has not been reported in studies on sickness absentees due to SRMD before. In younger individuals, risk markers related to the severity of the underlying disease (including mental inpatient care and long sickness absences) were more strongly associated with an increased risk of granted disability pension than among older individuals. Also if young individuals lived outside big cities and had a low educational level, they had a higher risk of being granted a disability pension during the five years of follow-up than their older counterparts. The associations in our study remained after adjustment of differences in specialized health care and a number of other socio-demographic factors. Socio-economic and regional differences in health care, work-place rehabilitation and labor market policies may be related to these findings and should be elucidated in future studies.

## Conclusions

Female sex, low education, increasing age, born outside Europe (EU25/Nordic countries), living in rural areas or alone, long duration of sickness absence, comorbidity with somatic disorders and severity of the underlying mental disorder increased the risk of granted DP in individuals with sick leave due to stress-related mental disorders. Some different patterns emerged for risk factors related to granted disability pension due to mental and somatic diagnoses and for younger and older individuals. The variation in the effect of risk markers with regard to age and diagnosis of disability pension speaks in favour of the importance of a person-centered approach in treatment and rehabilitation.
